# Coagulation parameters for the differential diagnosis of pancreatic cancer in the early stage: a retrospective study

**DOI:** 10.1186/s40001-023-01379-x

**Published:** 2023-10-17

**Authors:** Li Jiaao, Ge Wanli, Zhang Kai, Guo Feng, Peng Yunpeng

**Affiliations:** 1 Kangda College, Nanjing Medical University, 101 Longmian Road, Nanjing, 210000 Jiangsu People’s Republic of China; 2 Pancreas Center, First Affiliated Hospital of Nanjing Medical University, 300 Guangzhou Road, Nanjing, 210029 Jiangsu People’s Republic of China; 3 Pancreas Institute, Nanjing Medical University, Nanjing, 210029 Jiangsu People’s Republic of China

**Keywords:** Pancreatic cancer, Coagulation parameters, Diagnosis, Early diagnosis

## Abstract

**Background:**

In recent years, conventional coagulation (CC) and thromboelastography (TEG) parameters have been reported to be closely related to the progression of pancreatic cancer (PC). However, the potential utility of these parameters in differentiating benign and malignant pancreatic diseases is still unclear.

**Objectives:**

A retrospective study was conducted to evaluate the efficacy of coagulation parameters in differentiating pancreatic cancer/early stage pancreatic cancer (EPC, TNM stages I and II) from benign control conditions, and to further explore whether coagulation parameters could improve the differential value of CA199.

**Methods:**

Receiver operating characteristic (ROC) curves and logistic regression analysis were used to identify the diagnostic value of each coagulation parameter or combination of parameters.

**Results:**

Compared with benign pancreatic disease (BPD), patients with pancreatic malignant tumors had significant coagulation disorders, specifically manifested as abnormal increases or decreases in several CC and TEG parameters (such as activated partial thromboplastin time (APTT), fibrinogen (FIB), D-dimer (DD2), K time, R time, Angle, maximum amplitude (MA), coagulation index (CI), and Ly30). In the training group, ROC curve showed that FIB, DD2, Angle, MA, and CI had favorable efficacy at differentiating PC or EPC from BPD (for PC, AUC = 0.737, 0.654, 0.627, 0.602, 0.648; for EPC, AUC = 0.723, 0.635, 0.630, 0.614, 0.648). However, several combined diagnostic indicators based on FIB, DD2 and CI failed to outperform the individual coagulation indexes in diagnostic efficiency. Combinations of certain coagulation indexes with CA199 outperformed CA199 alone at identifying PC or EPC, especially FIB + CA199 (for PC, AUC = 0.904; for EPC, AUC = 0.905), FIB + DD2 + CA199 (for PC, AUC = 0.902; for EPC, AUC = 0.900), FIB + CI + CA199 (for PC, AUC = 0.906; for EPC, AUC = 0.906), and FIB + DD2 + CI + CA199 (for PC, AUC = 0.905; for EPC, AUC = 0.900). The results from a validation set also confirmed that these combinations have advantageous diagnostic value for PC and EPC.

**Conclusions:**

A significant hypercoagulable state was common in PC. Some CC and TEG parameters are valuable in the differential diagnosis of benign and malignant pancreatic diseases. In addition, coagulation indexes combined with CA199 can further enhance the differential diagnosis efficacy of CA199 in PC and EPC.

**Supplementary Information:**

The online version contains supplementary material available at 10.1186/s40001-023-01379-x.

## Introduction

Pancreatic cancer (PC) is one of the most common malignant tumors of the digestive system characterized by difficult early diagnosis, a low radical resection rate, high mortality, and a low 5-year survival rate (less than 10%) [1, 2]. In recent decades, a large number of studies have been performed to explore of the pathogenesis, progression mechanisms, surgery, and adjuvant therapy for pancreatic cancer. However, The diagnosis for early stage pancreatic cancer (EPC) is a particularly concerning challenge. [3–5]. The early diagnosis of PC is a particularly concerning challenge. According to the literature, the proportion of PC patients without local progression and/or distant metastasis at the time of diagnosis is only 20%, and the rest have no opportunity to undergo radical surgical resection [6, 7]. However, the tertiary prevention strategy of the International Anti-Cancer Alliance for malignant tumors suggested that a favorable therapeutic effect could be achieved by detecting tumors at an early stage [8]. Therefore, the key to improving the diagnosis and treatment status of PC is to explore efficient diagnosis methods and increase the diagnosis rate of EPC.

At present, CA199 is still the most commonly used biomarker for PC diagnosis. However, the specificity and the sensitivity of CA99 have some limitations [9, 10]. In recent years, to improve the diagnosis rate of EPC, researchers have made many efforts to screen for diagnostic markers. Previous studies have shown that many types of protein, RNA, circulating tumor cells (CTCs), circulating tumor DNA (ctDNA), cell-free DNA (cfDNA) and other peripheral blood indicators have individual or combined diagnostic value for PC, although the diagnostic efficacy of these indicators is markedly different. For example, a multicenter clinical study showed that MUC5AC (either alone or in combination with CA199) could effectively differentiate benign and malignant pancreatic tumors [11]. Exocrine-derived long RNA combinations (including FGA, KRT19, HIST1H2BK, ITIH2, MARCH2, CLDN1, MAL2 and TIMP1) could accurately distinguish PC from chronic pancreatitis [12]. A retrospective study showed that the combination of THBS2, CA199 and cfDNA could significantly improve the early diagnosis rate of PC compared with a single indicator [13]. However, due to some limitations (such as high cost, complex detection processes, and immature detection technology), newly discovered diagnostic markers are rarely used in clinical practice.

To avoid these limitations of novel indicators, some studies also evaluated the diagnostic value of existing clinical laboratory indicators for pancreatic cancer. In addition to CA199, some other tumor markers, such as CA125, CA242, and CEA, were evaluated to determine whether they could be used for PC diagnosis [14–16]. The results suggested that the diagnostic efficacy of these markers used alone was usually worse than that of CA199, but they could be applied for Lewis antigen-negative PC or combined with CA199 [17]. In addition, other indicators that differ between benign and malignant tumors, such as inflammatory indicators, metabolic parameters, and cell-free components, may also have diagnostic value for PC [18–20]. Among them, the role of coagulation indicators in cancer diagnosis and prognosis prediction has received extensive attention in recent years. Previous studies have reported that there are many abnormal coagulation parameters in the peripheral blood of patients with PC, some of which are closely associated with a poor prognosis [21, 22].

However, few studies focused on the roles of coagulation parameters in the diagnosis of PC. Therefore, the aim of our study is to comprehensively analyze the diagnostic value of coagulation parameters for PC and/or EPC by performing a retrospective study.

## Materials and methods

### Patients

Patients treated for pancreatic conditions in the Pancreatic Center of Jiangsu Province Hospital from June 2016 to September 2021 were retrospectively enrolled in this study, including 258 patients with PC and 102 patients with benign or borderline pancreatic disease (including serous cystadenoma, mucinous cystadenoma, intraductal papillary mucinous tumor, solid pseudopapillary tumor, G1/2 grade neuroendocrine tumor, and chronic pancreatitis). According to their pathological diagnosis, all patients were divided into a malignant disease group and a benign disease group. Borderline pancreatic diseases were classified as part of the benign disease group in this study. The detailed clinicopathological information of patients in each group is shown in Table 1.

**Table 1:** Basic information of enrolled patients

	Pancreatic Cancer	Benign Pancreatic Disease
Number	258	102
Age (Median, Q1–Q3)	64, 58–70	54, 42–65
Gender		
Male	164	50
Female	94	52
Pathology distribution	PADC (249)	MCN (11)
	PASC (6)	SCN (27)
	Other (3)	IPMN (26)
		NET (10)
		SPT (10)
		CP (18)

PADC represents pancreatic ductal adenocarcinoma; PASC represents pancreatic adenosquamous carcinoma; SCN represents serous cystadenoma; MCN represents mucinous cystadenoma; IPMN represents intraductal papillary mucinous tumor; SPT represents solid pseudopapillary tumor; NET represents neuroendocrine tumor; CP represents chronic pancreatitis

All included patients had a definitive pathological diagnosis and complete coagulation data. Patients with the following situations were excluded: lack of detailed clinical and pathological data, lack of coagulation data, co-occurrence of other malignant tumors, co-occurrence of active inflammatory diseases, and co-occurrence of hematological diseases. This study was approved by the Ethics Committee of Jiangsu Provincial People’s Hospital.

### Data collection

The clinical and pathological information required for this study was prospectively collected and archived in the clinical database of Pancreatic Center of Jiangsu Provincial Hospital, as well as coagulation index data. Clinical and pathological data applied in this study included gender, age, preoperative CA199, and pathological data; preoperative conventional coagulation (CC) indexes included thrombin time (TT), activated partial thromboplastin time (APTT), prothrombin time (PT), fibrinogen (FIB), D-dimer (DD2), and platelet count (PLT); and thromboelastography (TEG)-related parameters included R time, K time, Angle, maximum amplitude (MA), coagulation index (CI), and LY30. TT, APTT, PT, FIB, and DD2 were detected by coagulation analyzer (Sysmex, Kobe, Japan), PLT was detected by blood cell counter (Sysmex, Kobe, Japan), and TEG was detected by Thromboelastography Analyzer (Haemonetics Corporation, Boston, USA). All data were rechecked by two researchers independently after collection.

### Statistical analysis

The Mann–Whitney* U* test and independent-samples *T* test were, respectively, used for testing the difference between continuous data with non-normal distribution and normal distribution; Chi-square test was used to test for significant differences in categorical variables between two groups (SPSS statistics Version 27, IBM, Chicago, USA). A receiver operating characteristic (ROC) curve was plotted, and the cutoff value, sensitivity, specificity, and area under the ROC curve (AUC) of coagulation variables were calculated (RStadio Desktop, Poist, Boston, USA). AUC between 0.5 and 0.6 suggests bad accuracy of the diagnostic test, between 0.6 and 0.7 suggests sufficient accuracy, between 0.7 and 0.8 good accuracy, between 0.8 and 0.9 very good accuracy, whereas AUC higher than 0.9 suggests an excellent accuracy. Logistic regression analysis was used to evaluate the combined diagnostic value of coagulation indicators (RStadio Desktop, Poist, Boston, USA). *P* value less than 0.05 was defined as statistically significant.

## Results

### Differences in coagulation parameters between patients with benign and malignant pancreatic disease

**Fig. 1:**
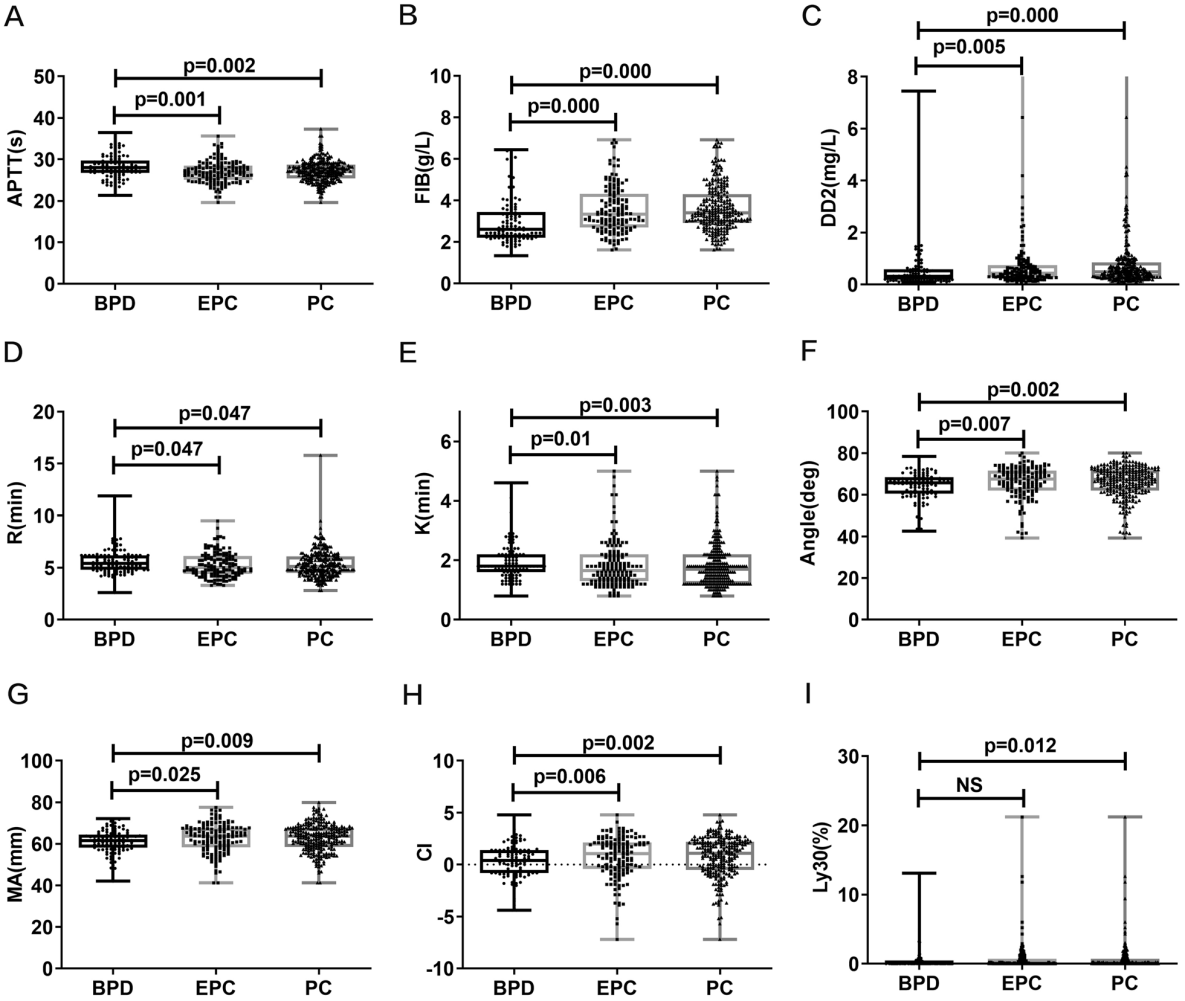
Differences of coagulation parameters between patients with benign and malignant pancreatic diseases. **A**–**I** Differences of coagulation parameters (APTT, FIB, DD2, R, K, Angle, MA, CI, and Ly30) between patients with pancreatic cancer/early stage pancreatic cancer and benign diseases. APTT represents activated partial thromboplastin time, FIB represents fibrinogen, DD2 represents d-dimer, R represents reaction time, K represents kinetics of clot development time, MA represents maximum amplitude, CI represents coagulation index, PC represents pancreatic cancer, EPC represents early stage pancreatic cancer, and BPD represents benign pancreatic disease

To evaluate whether coagulation parameters are useful for distinguishing benign and malignant pancreatic disease, we first compared each parameter between the benign and malignant groups. The analytic results showed that there were abnormal alterations in some CC and thromboelastography (TEG) parameters in the malignant tumor group, specifically manifested as decreased APTT and R time and increased FIB, DD2, MA, Angle, and CI. Further comparison of these parameters between EPC and BPD revealed that APTT, FIB, DD2, R time, MA, Angle, and CI were also significantly increased or decreased in the EPC group (Table 2, Fig. 1). All results in this aspect suggested that these CC and TEG parameters have the potential to differentiate benign and malignant pancreatic disease, as well as the potential function to identify EPC from BPD.

**Table 2:** Differences of coagulation parameters in cancer and control groups

	PC		BPD		*P*	EPC		BPD		*P*
	Median (Mean)	Q1–Q3 (SD)	Median (Mean)	Q1–Q3 (SD)		Median (Mean)	Q1–Q3 (SD)	Median (Mean)	Q1–Q3 (SD)	
PT(s)	11.90	11.48–12.33	11.70	11.30–12.30	0.393	11.70	11.30–12.20	11.70	11.30–12.30	0.656
APTT(s)	27.19	2.59	28.25	2.86	0.001	26.97	2.67	28.25	2.86	0.000
FIB(g/L)	3.395	2.92–4.29	2.61	2.21–3.44	0.000	3.33	2.70–4.31	2.61	2.21–3.44	0.000
TT(s)	17.30	16.68–18.1	17.7	16.78–18.30	0.059	17.50	16.80–18.10	17.7	16.78–18.30	0.354
DD2(mg/L)	0.49	0.29–0.85	0.315	0.19–0.58	0.000	0.44	0.28–0.74	0.315	0.19–0.58	0.005
PLT(10^9/L)	186.00	144.75–236.00	195.50	156.00–232.00	0.352	187.50	148.00–238.25	195.50	156.00–232.00	0.496
R(min)	5.10	4.50–6.10	5.40	4.80–6.20	0.047	5.00	4.40–6.13	5.40	4.80–6.20	0.047
K(min)	1.70	1.20–2.20	1.80	1.60–2.20	0.003	1.65	1.30–2.20	1.80	1.60–2.20	0.010
Angle(deg)	67.30	62.0–71.75	65.60	60.50–68.33	0.002	67.55	61.95–71.48	65.60	60.50–68.33	0.007
MA(mm)	63.70	58.58–67.53	61.55	58.13–64.43	0.009	63.75	58.38–67.50	61.55	58.13–64.43	0.025
CI	1.10	− 0.50–2.20	0.40	− 0.83–1.40	0.002	1.05	− 0.43–2.13	0.40	− 0.83–1.40	0.006
Ly30(%)	0.00	0.00–0.70	0.00	0.00–0.20	0.012	0.00	0.00–0.73	0.00	0.00–0.20	0.058

*PC* represents pancreatic cancer, *EPC* represents early stage pancreatic cancer, *BPD* represents benign pancreatic disease. The continuous data with non-normal distribution has been presented as median (Q1–Q3), whereas continuous data with normal distribution has been presented as mean ± SD

Furthermore, we also analyzed the correlation between PC clinicopathological factors and APTT, as well as FIB, DD2, R, K, MA, Angle, CI, and LY30. The results revealed a significant association between DD2 and PC progression; specifically, the level of DD2 was further increased in patients with larger tumors and higher TNM stages (Additional file 4: Table S1).

### Value of coagulation parameters in identifying malignant pancreatic diseases

To further assess and verify the specific value of coagulation parameters in differentiating benign and malignant pancreatic disease, we divided all patients into a training set (the first 2/3 of patients) and a validation set according to the time of hospitalization. In the training set, we first evaluated the differential value of a single coagulation index using an ROC curve. As shown in Table 3, FIB, DD2, MA, Angle, and CI could effectively discriminate benign and malignant pancreatic disease, of which FIB had the highest value (AUC = 0.737), while DD2, Angle, MA, and CI had similar values (AUC = 0.654, 0.627, 0.602, and 0.648, respectively); moreover, the analytic results suggested that FIB, DD2, Angle, MA, and CI also had similar functions in differentiating EPC and benign control conditions, and the AUCs of these parameters were 0.723, 0.635, 0.630, 0.614, and 0.648, respectively.

**Table 3:** Differential value of coagulation indexes for PC based on ROC curve

	Optimal Cutpoint	Sensitivity	Specificity	PPV	NPV	Positive LR	Negative LR	AUC	Accuracy
(1)									
PT(s)	12.85	0.912	0.162	0.731	0.423	1.088	0.545	0.506	0.697
APTT(s)	28.25	0.747	0.500	0.789	0.442	1.494	0.506	0.666	0.676
FIB(g/L)	2.7	0.841	0.618	0.846	0.609	2.200	0.257	0.737	0.777
TT(s)	17.65	0.488	0.676	0.790	0.346	1.509	0.757	0.587	0.542
DD2(mg/L)	0.235	0.835	0.426	0.785	0.509	1.456	0.386	0.654	0.718
PLT(10^9/L)	244.5	0.241	0.838	0.788	0.306	1.491	0.905	0.494	0.412
R(min)	5.05	0.482	0.794	0.854	0.380	2.343	0.652	0.628	0.571
K(min)	1.65	0.447	0.779	0.835	0.361	2.027	0.709	0.619	0.542
Angle(deg)	68.55	0.412	0.838	0.864	0.363	2.545	0.702	0.627	0.534
MA(mm)	66.95	0.312	0.912	0.898	0.346	3.533	0.755	0.602	0.483
CI	1.55	0.388	0.897	0.904	0.370	3.772	0.682	0.648	0.534
LY30(%)	0.15	0.429	0.750	0.811	0.345	1.718	0.761	0.575	0.521

	Optimal Cutpoint	Sensitivity	Specificity	PPV	NPV	Positive LR	Negative LR	AUC	Accuracy
(2)									
PT(s)	11.25	0.248	0.868	0.743	0.428	1.871	0.867	0.562	0.491
APTT(s)	26.75	0.505	0.779	0.779	0.505	2.288	0.635	0.671	0.613
FIB(g/L)	2.7	0.790	0.618	0.761	0.656	2.067	0.339	0.723	0.723
TT(s)	17.85	0.571	0.588	0.682	0.471	1.388	0.729	0.565	0.578
DD2(mg/L)	0.255	0.790	0.471	0.697	0.593	1.493	0.445	0.635	0.665
PLT(10^9/L)	244.5	0.248	0.838	0.703	0.419	1.531	0.898	0.526	0.480
R(min)	5.05	0.486	0.794	0.785	0.500	2.359	0.648	0.621	0.607
K(min)	1.65	0.467	0.779	0.766	0.486	2.116	0.684	0.621	0.590
Angle(deg)	68.55	0.448	0.838	0.810	0.496	2.767	0.659	0.630	0.601
MA(mm)	65.35	0.429	0.824	0.789	0.483	2.429	0.694	0.614	0.584
CI	1.55	0.410	0.897	0.860	0.496	3.978	0.658	0.648	0.601
LY30(%)	0.15	0.390	0.750	0.707	0.443	1.562	0.813	0.554	0.532

*PPV* represents positive predictive value, *NPV* represents Negative Predictive Value. LR represents likelihood ratio, *AUC* represents Area Under Curve

Then, we investigated the combined performance of the coagulation indexes mentioned above. Due to the remarkable correlations between three TEG parameters (including MA, Angle and CI), we selected only CI for subsequent analysis because of its high diagnostic efficiency (Additional file 1: Figure S1). For the differentiation of PC and BPD, FIB + CI was the best diagnostic combination (AUC = 0.745), followed by FIB + DD2 and DD2 + CI (AUC = 0.736, 0.656); for the diagnosis of EPC and BPD, FIB + CI had the highest diagnostic efficacy, followed by FIB + DD2 and DD2 + CI (AUC = 0.729, 0.715, and 0.650, respectively) (Table 4, Additional file 2: Figure S2). However, the combined performance of the two coagulation indicators did not significantly increase compared with FIB in either PC vs. BPD or EPC vs. BPD. In addition, the differential efficacy of combination with FIB, DD2, and CI was also similar to that of FIB (for PC, AUC = 0.745; for EPC, AUC = 0.721).

**Table 4:** Combined diagnostic efficiency of coagulation indexes

	PC vs. BPD			EPC vs. BPD		
	Sensitivity	Specificity	AUC	Sensitivity	Specificity	AUC
DD2 + CI	0.853	0.465	0.656	0.897	0.410	0.650
DD2 + FIB	0.618	0.841	0.736	0.632	0.762	0.715
FIB + CI	0.691	0.806	0.745	0.721	0.724	0.729
DD2 + FIB + CI	0.691	0.806	0.745	0.750	0.667	0.721

*PC* represents pancreatic cancer, *EPC* represents early stage pancreatic cancer, *BPD* represents benign pancreatic disease, *AUC* represents Area Under Curve

### Value of coagulation parameters combined with CA199 in differentiating malignant pancreatic diseases

CA199 is the most common serum biomarker for the diagnosis and differential diagnosis of PC in clinical practice. Therefore, we also calculated the efficacy of CA199 based on data from the training set (AUC = 0.851 for BPD vs. PC; AUC = 0.848 for BPD vs. EPC). To explore whether FIB, DD2, CI, and their combinations could further improve the differential efficiency of CA199, we analyzed the diagnostic AUC of CA199 combined with a single indicator, two indicators, and three indicators. The related results are shown in Table 5 and Additional file 3: Figure S3. For the differential diagnosis of PC or EPC, some combinations of coagulation indicators and CA199 improved somewhat on the diagnostic value of CA199, especially CA199 + FIB (AUC = 0.904 for BPD vs. PC; AUC = 0.905 for BPD vs. EPC), CA199 + DD2 + FIB (AUC = 0.902 for BPD vs. PC; AUC = 0.900 for BPD vs. EPC), CA199 + CI + FIB (AUC = 0.906 for BPD vs. PC; AUC = 0.906 for BPD vs. EPC), and CA199 + CI + FIB + DD2 (AUC = 0.905 for BPD vs. PC; AUC = 0.900 for BPD vs. EPC).

**Table 5:** Combined diagnostic efficiency of coagulation indexes and CA199

	PC vs. BPD			EPC vs. BPD		
	Sensitivity	Specificity	AUC	Sensitivity	Specificity	AUC
DD2 + CA199	0.882	0.829	0.892	0.882	0.819	0.885
FIB + CA199	0.882	0.835	0.904	0.882	0.829	0.905
CI + CA199	0.897	0.824	0.885	0.853	0.857	0.880
DD2 + FIB + CA199	0.868	0.847	0.902	0.824	0.895	0.900
DD2 + CI + CA199	0.897	0.829	0.887	0.897	0.810	0.881
FIB + CI + CA199	0.912	0.818	0.906	0.912	0.800	0.906
DD2 + FIB + CI + CA199	0.912	0.818	0.905	0.853	0.857	0.900

*PC* represents pancreatic cancer, *EPC* represents early stage pancreatic cancer, *BPD* represents benign pancreatic disease, *AUC* represents Area Under Curve

### Performance of coagulation parameters combined with CA199 in the validation set

**Fig. 2:**
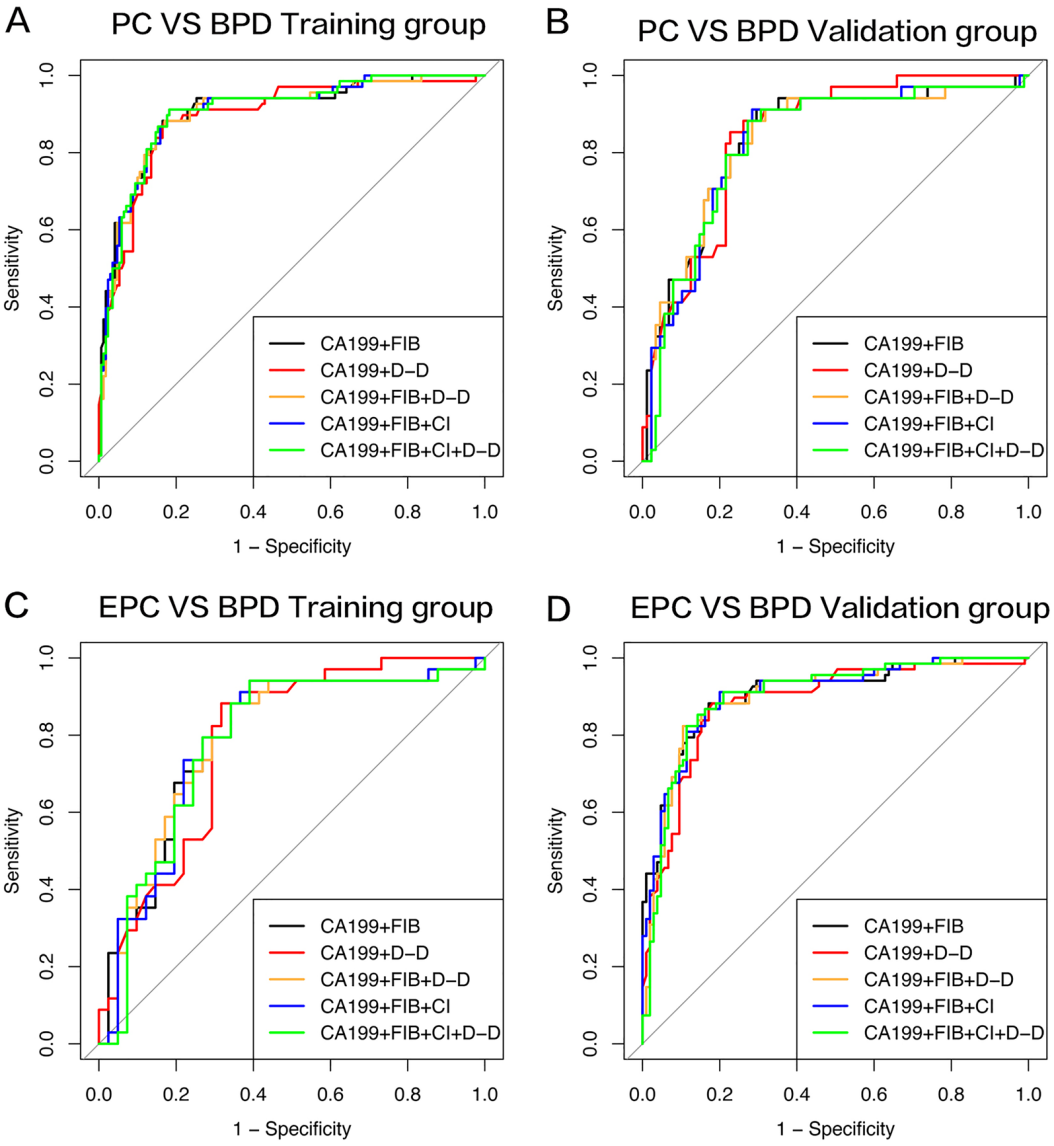
Diagnostic perform of coagulation parameters combined with CA199. **A**, **B** ROC curves of coagulation parameters or their combination with CA199 for distinguishing pancreatic cancer from benign pancreatic diseases in training and validation group. **C**, **D** ROC curves of these indicators with CA199 for identifying early stage pancreatic cancer from benign pancreatic diseases in training and validation group. D–D represents DD2

To clarify the value of CA199 + FIB and CA199 + CI + FIB for the differential diagnosis of benign and malignant pancreatic disease, we further verified these combinations on the validation set. For PC vs. BPD, the validation AUCs of CA199 + FIB, CA199 + DD2 + FIB, CA199 + CI + FIB, and CA199 + CI + FIB + DD2 were 0.838, 0.833, 0.829, and 0.825, respectively; for EPC vs. BPD, the AUCs of CA199 + FIB, CA199 + DD2 + FIB, CA199 + CI + FIB, and CA199 + CI + FIB + DD2 were 0.792, 0.786, 0.787, and 0.781, respectively (Fig. 2). In summary, the addition of FIB or FIB + CI could effectively improve the performance of CA199 in the diagnosis and early diagnosis of PC/EPC.

## Discussion

In this study, we comprehensively analyzed the differences in CC and TEG indicators in benign and malignant pancreatic disease and found that some indicators have the potential ability to differentiate PC. Among them, FIB, DD2, MA, Angle, and CI had satisfying efficacy in the diagnosis and early diagnosis of PC. However, the combination of two or three coagulation indicators did not outperform individual indicators in the diagnosis of PC or EPC. Furthermore, we found that the combination of CA199 and coagulation indicators could significantly enhance the early diagnostic performance of CA199.

In recent years, the relationship between abnormal coagulation status and various malignancies has gradually been uncovered, including the diagnostic and prognostic values of abnormal coagulation indicators for malignant tumors and the potential mechanisms of coagulation indicators in promoting tumor progression. Most previous studies have focused on the correlation between CC indicators and the prognosis of malignant tumors. For example, DD2 combined with the international normalized ratio (INR) could effectively predict poor prognosis in epithelial ovarian cancer, especially in the advanced stage [23]; the coagulation index score calculated based on PLT, mean platelet volume (MPV), and FIB was an independent risk factor for the prognosis of esophageal squamous cell carcinoma [24]; an abnormal increase in serum FIB was closely related to reduced disease-free and overall survival time in locally advanced PC [22]. In addition, the use of coagulation disorder in the diagnosis of some malignant tumors has also been reported. For example, APTT and platelet distribution width (PDW used individually or in combination showed good predictive values for nasopharyngeal carcinoma, while APTT, FIB, and DD2 were significantly associated with nasopharyngeal carcinoma metastasis [25]; there was an independent correlation between abnormally increased FIB and prostate cancer sensitivity [26]; serum DD2 was a potential marker for the diagnosis of gallbladder cancer, and DD2 combined with CA199 had excellent diagnostic value for this cancer (AUC = 0.920) [27]. As in other malignant tumors, some CC parameters have been reported to have diagnostic value for PC. The serum FIB level in patients with PC was significantly higher than that in healthy controls, and the FIB level in metastatic PC patients was further increased [28]; compared to patients with low-risk intraductal papillary mucinous neoplasms (IPMN), those with high-risk IPMN had a markedly elevated level of FIB [29]; most PC patients suffered from abnormal coagulation alteration at the time of diagnosis, and tissue factor (TF) and thrombin–antithrombin (TAT) had potential roles in differentiating metastatic PC [30]. Similar to previous studies, this study also found that some CC indicators, namely, APTT, FIB, and DD2, were abnormally altered in patients with PC compared with patients with BPD; among them, FIB and DD2 could efficiently differentiate PC and EPC according the ROC results.

In addition to CC indicators, TEG is also widely used in the clinical monitoring of coagulation and fibrinolysis function, and its relationships with malignant tumors are also being explored. For example, the TEG parameters K time, Angle, and MA can predict the stage of lung cancer [31]. It has also been reported that advanced colorectal cancer usually exhibits hypercoagulable status, and MA is a potential effective biomarker for the identification of such cancer [32]. In addition, TEG parameters were significantly abnormal in patients with thyroid cancer; among these parameters, Angle, CI, and thrombodynamic potential index (TPI) had potential diagnostic utility for this type of cancer [33]. However, relatively few articles have explored the associations between TEG and PC development. Previous studies showed that many TEG parameters were abnormal in patients with PC, and the abnormal parameters were significantly correlated with tumor type, nodular disease, and tumor resectability [34]. Angle might be an effective target for predicting early recurrence, disease-free survival time, and overall survival time in PC [35]. Similar to previous studies, our study also showed that multiple TEG parameters were markedly different between benign and malignant pancreatic disease, including Angle, MA, and CI. In further analysis, we reported the novel finding that Angle, MA, and CI have favorable diagnostic efficacy for both PC and EPC. In addition, the analytic data showed that the diagnostic efficacy of TEG parameters for PC was similar to that of CC parameters. We also analyzed the combined diagnostic value of some coagulation parameters (FIB, DD2, and CI) in further investigation. However, two or three combinations of these coagulation indicators could not effectively improve the diagnostic efficacy for PC and EPC.

Previous studies focusing on PC diagnostic markers usually evaluated whether the combination of investigated targets and CA199 could further improve diagnostic efficiency for PC. For example, Rahat Jahan et al. reported that the combined diagnosis of trefoil factors and CA199 could further increase the sensitivity and specificity of CA199 in the diagnosis of EPC [36]; Jiayu Zhang and Sukhwinder Kaur both found that MUC5AC combined with CA199 has extremely high diagnostic value for PC [11, 37]. In this study, we conducted similar analyses based on our training set and validation set, and the analytic results revealed that some coagulation indicators or their combinations could significantly improve the value of CA199 for the differential diagnosis of PC and EPC. Among these, CA199 + FIB and CA199 + CI + FIB were the most effective combinations. We also performed similar analyses in this study. In the training set, the analytic results revealed that some coagulation indicators or their combinations could significantly improve the value of CA199 for the differential diagnosis of PC and EPC, including CA199 + FIB, CA199 + DD2 + FIB, CA199 + CI + FIB, and CA199 + CI + FIB + DD2. The results based on validation set further confirmed the findings of training group, especially the diagnostic performance of CA199 + FIB and CA199 + FIB + CI for PC and EPC.

Of course, this study also had some limitations. First, healthy volunteers were not included in the control group, so the results obtained from existing data were applicable for the differential diagnosis of benign and malignant pancreatic disease rather than PC screening. Second, this study was a retrospective study with a relatively insufficient sample size; thus, the level of evidence was also limited. In addition, this study used only the data from our center to preliminarily verify the results in the training group; there was no validation on external data. In summary, a multicenter, prospective randomized controlled study is urgently needed to verify the findings of this study.

## Conclusion

Abnormally altered FIB, DD2, MA, Angle, and CI could effectively differentiate PC and EPC from BPD. CA199 + FIB, CA199 + DD2 + FIB, CA199 + CI + FIB, and CA199 + CI + FIB + DD2 could provide significantly better diagnostic value for PC and EPC, especially CA199 + FIB and CA199 + FIB + CI. The identification of effective diagnostic markers or combinations will improve the early diagnosis of PC.

### Supplementary Information

**Additional file 1: Figure S1**. Association between MA, CI, and Angle. (A) Association between MA and Angle in pancreatic cancer patients. (B) Association between MA and CI in pancreatic cancer patients. (C) Association between CI and Angle in pancreatic cancer patients.

**Additional file 2: Figure S2**. Diagnostic perform of coagulation parameter combinations in training group. **A–D** ROC curves of different coagulation parameter combinations (DD2+CI, DD2+FIB, FIB+CI, and DD2+FIB+CI) for distinguishing pancreatic cancer from benign pancreatic diseases. **E–H** ROC curves of these combinations for distinguishing early stage pancreatic cancer from benign pancreatic diseases.

**Additional file 3: Figure S3**. Diagnostic perform of coagulation parameters combined with CA199 in training group. **A–G** ROC curves of different coagulation parameters or their combinations with CA199 (CA199+FIB, CA199+DD2, CA199+CI, CA199+FIB+CI, CA199+DD2+FIB, CA199+DD2+CI, and CA199=DD2+FIB+CI) for distinguishing pancreatic cancer from benign pancreatic diseases. **H–N** ROC curves of these combinations for distinguishing early stage pancreatic cancer from benign pancreatic diseases.

**Additional file 4: Table S1**. The association between PC clinicopathological factors and coagulation parameters.

## Data Availability

All data applied in this study were provided in related files.

## Abbreviations

PC: Pancreatic cancer
EPC: Early stage pancreatic cancer
BPD: Benign pancreatic disease
ROC curve: Receiver operating characteristic curve
FIB: Fibrinogen
DD2: D-dimer
MA: Maximum amplitude
CI: Coagulation index
CA199: Cancer antigen 199
AUC: Area under the ROC curve
CC: Conventional coagulation
TEG: Thromboela-stogram
CTCs: Circulating tumor cells
ctDNA: Circulating tumor DNA
cfDNA: Cell-free DNA
MUC5AC: Mucin 5AC
CA125: Cancer antigen 125
CA242: Cancer antigen 242
CEA: Carcino-embryonic antigen
TT: Thrombin time
APTT: Activated partial thromboplastin time
PT: Prothrombin time
PLT: Platelet count
R time: Reaction time
K time: Kinetics of clot development time
INR: International normalized ratio
MPV: Mean platelet volume
IPMN: Intraductal papillary mucinous neoplasms
TF: Tissue factor
TAT: Thrombin–antithrombin
TPI: Thrombodynamic potential index
PADC: Pancreatic ductal adenocarcinoma
PASC: Pancreatic adenosquamous carcinoma
SCN: Serous cystadenoma
MCN: Mucinous cystadenoma
SPT: Solid pseudopapillary tumor
NET: Neuroendocrine tumor
CP: Chronic pancreatitis
