# Supplementary figures and images for: Coagulation parameters for the differential diagnosis of pancreatic cancer in the early stage: a retrospective study
Source: Eur J Med Res. 2023 Oct 17;28:436. doi: 10.1186/s40001-023-01379-x (PMC10580648; doi:10.1186/s40001-023-01379-x)

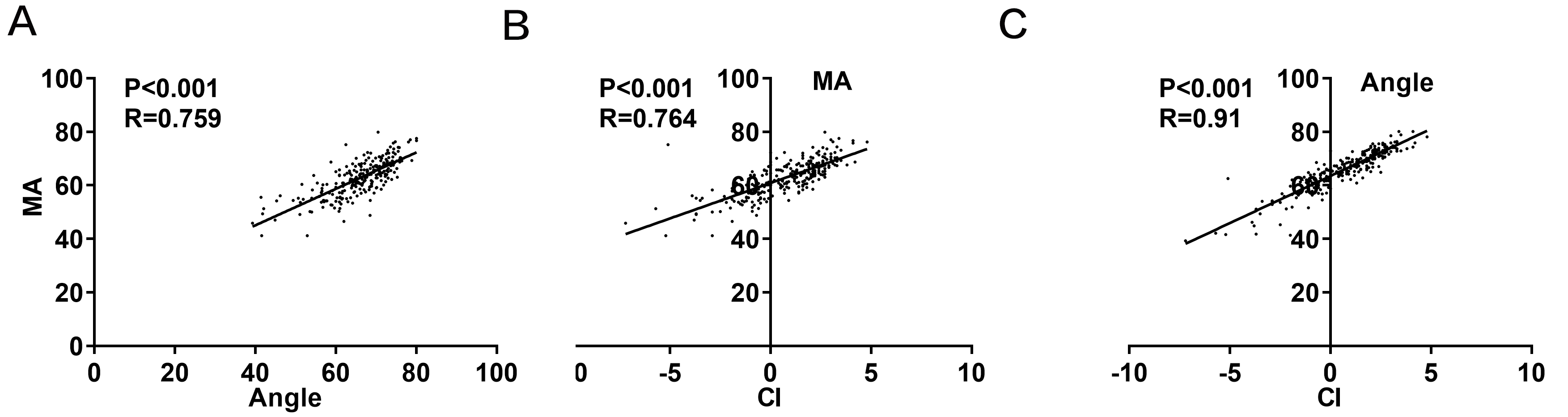

Supplement: Supplementary file 1 — Additional file 1: Figure S1. Association between MA, CI, and Angle. (A) Association between MA and Angle in pancreatic cancer patients. (B) Association between MA and CI in pancreatic cancer patients. (C) Association between CI and Angle in pancreatic cancer patients. [file 40001_2023_1379_MOESM1_ESM.jpg]
