# Supplementary material for: Coagulation parameters for the differential diagnosis of pancreatic cancer in the early stage: a retrospective study
Source: Eur J Med Res. 2023 Oct 17;28:436. doi: 10.1186/s40001-023-01379-x (PMC10580648; doi:10.1186/s40001-023-01379-x)
Supplement: Supplementary file 4 — Additional file 4: Table S1. The association between PC clinicopathological factors and coagulation parameters. [file 40001_2023_1379_MOESM4_ESM.docx]

| Table S1. The association between PC clinicopathological factors and coagulation parameters | | | | | | | | | | | | | | | |
| --- | --- | --- | --- | --- | --- | --- | --- | --- | --- | --- | --- | --- | --- | --- | --- |
|  | CA199 low | | CA199 high | | *P* | Size≤4cm | | Size＞4cm | | *P* | TNM=I/II | | TNM=III/IV | | *P* |
|  | Mean | SD | Mean | SD |  | Mean | SD | Mean | SD |  | Mean | SD | Mean | SD |  |
| APTT(s) | 26.96 | 2.55 | 27.25 | 2.60 | 0.664 | 27.29 | 2.62 | 27.02 | 2.54 | 0.563 | 26.97 | 2.66 | 27.47 | 2.47 | 0.073 |
| FIB(g/L) | 3.48 | 1.08 | 3.67 | 1.10 | 0.204 | 3.58 | 1.09 | 3.73 | 1.10 | 0.300 | 3.56 | 1.13 | 3.72 | 1.04 | 0.119 |
| DD2(mg/L) | 0.98 | 1.73 | 0.93 | 1.79 | 0.515 | 0.74 | 1.41 | 1.29 | 2.23 | 0.001 | 0.83 | 1.57 | 1.10 | 2.01 | 0.015 |
| R(min) | 5.33 | 1.25 | 5.32 | 1.35 | 0.945 | 5.47 | 1.42 | 5.07 | 1.12 | 0.017 | 5.28 | 1.19 | 5.37 | 1.49 | 0.615 |
| K(min) | 1.93 | 0.81 | 1.78 | 0.72 | 0.206 | 1.78 | 0.70 | 1.87 | 0.81 | 0.512 | 1.84 | 0.78 | 1.79 | 0.70 | 0.768 |
| Angle(deg) | 64.98 | 7.97 | 66.47 | 7.29 | 0.196 | 66.55 | 6.87 | 65.53 | 8.31 | 0.446 | 66.00 | 7.73 | 66.39 | 7.07 | 0.858 |
| MA(mm) | 61.73 | 6.83 | 63.18 | 6.62 | 0.110 | 63.24 | 6.55 | 62.28 | 6.87 | 0.185 | 62.66 | 6.94 | 63.17 | 6.32 | 0.777 |
| CI | 0.48 | 2.08 | 0.83 | 1.88 | 0.311 | 0.75 | 1.92 | 0.79 | 1.95 | 0.790 | 0.73 | 2.03 | 0.80 | 1.79 | 0.936 |
| Ly30(%) | 0.60 | 1.71 | 0.72 | 2.00 | 0.333 | 0.78 | 2.33 | 0.56 | 1.00 | 0.955 | 0.80 | 2.38 | 0.56 | 1.15 | 0.509 |
